# Predictors and shared traits of longevity within 1 year before death in patients with schizophrenia receiving long-term care: 3-year retrospective cross-sectional study

**DOI:** 10.1192/bjo.2024.796

**Published:** 2024-10-08

**Authors:** Chuan-Hsun Yu, Tsung-Cheng Hsieh

**Affiliations:** Department of General Psychiatry, Yuli Hospital, Ministry of Health and Welfare, Hualien, Taiwan; Institute of Medical Sciences, Tzu-Chi University, Hualien, Taiwan

**Keywords:** Life expectancy, mortality and morbidity, psychotic disorders/schizophrenia, mental health services, old age psychiatry

## Abstract

**Background:**

Research on schizophrenia and life expectancy has mainly focused on premature mortality.

**Aims:**

This study investigates factors associated with longevity in patients with schizophrenia receiving long-term care and identifies shared traits among these individuals.

**Method:**

A retrospective cross-sectional study analysing the clinical records of 138 patients with schizophrenia who died between 2015 and 2017 in a psychiatric long-term care facility was conducted. Longevity was defined by life tables drawn from the national health database. Variables were compared between longevity and control groups to determine predictors of longer lifespans. Cluster analysis was employed to identify shared traits among individuals with longevity. Causes of death by age were compared.

**Results:**

In the long-term care setting, of the 138 participants, 45 were in the longevity group. This group had more males, lower antipsychotic doses, but more mobility issues. Significant predictors of longevity included older age at onset, longer length of stay, lower activities of daily living scores and a hypertension diagnosis. Cluster analysis revealed two patterns, suggesting that poorer health indicators did not necessarily lead to shorter lives. Fatalities caused by pneumonia were associated with a higher age, compared to those from cancer and choking.

**Conclusions:**

Addressing modifiable risk factors enhances life expectancy in patients with schizophrenia, especially for males, while the age at onset may play a significant role. An integrated long-term care model with close monitoring and timely provision of mental and general healthcare may help extend lifespans. Further research is needed to balance long-term residential care and community-based care for elderly patients with schizophrenia.

Schizophrenia is a chronic and debilitating mental illness that can significantly impair an individual's cognitive and physical ability to function in daily life.^1^ Patients with schizophrenia are also at increased risk for physical comorbidities, such as cardiovascular disease, diabetes, obesity and pneumonia.^2,3^ These conditions are often linked to lifestyle factors such as poor diet, lack of physical activity, smoking, substance misuse and the adverse effects of antipsychotic medications on metabolic functioning.^4^ The mortality rate in people with schizophrenia is more than 3.5 times higher than that of the general population, and this excess mortality is largely because of treatable physical health conditions, including diabetes, cardiovascular disease, respiratory disease and other natural causes.^5^ Along with the previously mentioned factors, age, gender, comorbidities and psychotropic medication use are known to affect the life expectancy of schizophrenia sufferers.^6–8^

Owing to medical advances and improved healthcare, life expectancy has increased faster among individuals with a history of common diseases.^9^ This medical success diverges when applied to people with schizophrenia, who have an augmented standardised mortality ratio compared to the general population.^10^ The mortality gap between people with schizophrenia and the general population is estimated to be 15–20 years^11,12^ and has widened in the past 30–40 years, highlighting the need for changes in social stigmatisation, healthcare and economic policy.^10^ Approaches targeting modifiable factors, such as substance misuse, antipsychotic treatments and appropriate medication use, have been suggested to reduce the mortality gap.^13^ Interventions focusing on physical health conditions, such as the promotion of lifestyle changes and the provision of comprehensive physical healthcare, may be effective in reducing the excess mortality observed in schizophrenia. With ageing, the significance of physical requirements becomes more pronounced compared to mental health needs. There is an evident necessity for the implementation of new models of care in which mental health and general healthcare systems collaborate to address the evolving demands of these patients.^14^

The average lifetime prevalence of schizophrenia is just under 1%.^15^ The period prevalence of schizophrenia in Taiwan is about 0.6%.^16^ Heterogeneity of outcomes is common in schizophrenia as it progresses with age, suggesting the need for targeted treatment strategies tailored to different outcome categories in ageing individuals.^17^ Many treatment-refractory patients who would benefit from ongoing treatment receive long-term care in high-support residential settings in Taiwan. Individuals under long-term care have professional support for their daily needs and easier access to health monitoring and treatment. Consequently, the factors affecting their lifespan and resulting impact may differ from those of non-long-term care patients. Previous research on the life expectancy of patients with schizophrenia has focused mainly on premature death. Few studies have discussed the effectiveness of interventions to improve longevity in schizophrenia. Therefore, the study's primary objective was to identify real-world factors associated with longevity in patients with schizophrenia receiving long-term care. The secondary objective was to investigate shared traits among individuals with longevity.

## Method

### Study design and clinical data source

The Taiwan National Health Insurance (NHI) programme has merged major social insurance programmes since 1995. It provides universal health coverage for residents and offers reliable population health data. All patients with schizophrenia with registered catastrophic illnesses are enrolled in the NHI programme. The Taiwan National Health Insurance Research Database (NHIRD) contains comprehensive medical records and encrypted personal information, including age at death, for every resident of Taiwan.^18^ Based on the life tables drawn from NHIRD in each year of 2015–2017, longevity was defined as a lifespan at or beyond the age corresponding to the 90th survival percentile specific to all contemporary patients with schizophrenia.^19^ Gender-specific age cut points for longevity groups are shown in Table 1.

**Table 1:** Gender-specific age cut points corresponding to the 90th survival percentile in each year of 2015–2017

Year	Age cut points	
	Male	Female
2015	75	79.5
2016	73	81
2017	75	83

This is a retrospective cross-sectional study using the clinical records of adult patients that resided in a psychiatric long-term care facility in Taiwan. This facility is affiliated with a psychiatric hospital established by the government and provides long-term care services for treatment-resistant severe mental illness patients throughout Taiwan. The hospital offers psychiatric and general medical care through a team of certified psychologists, psychiatrists, family physicians, a neurologist and an internist specialising in endocrinology. Between 2015 and 2017, the average number of residents admitted was 2368 (male:female ratio of 2:1), and more than 90% of patients were diagnosed with schizophrenia, with a mean age of 52.8 years old. The study sample consisted of patients with schizophrenia who died between 2015 and 2017 at the psychiatric facility. After excluding 30 homeless individuals with unknown ages and cases with indeterminate age-related variables, a total of 138 patients covering all causes of mortality were enrolled. The 138 patients with schizophrenia were classified as the longevity group (*N* = 45) and the control group (*N* = 93), correspondingly.

### Study variables

Variables comprised sociodemographic information, comorbidities, clinical characteristics and causes of death. Sociodemographic information included: gender, age at onset, length of stay and age at death. The length of stay was calculated as the period from the initial admission to the facility until the time of death. Medical comorbidities were diagnosed by general medical clinicians and assessed by the Charlson comorbidity index (CCI).^20^ The CCI is a tool used to predict the 10-year mortality of a patient who has multiple comorbid conditions. It helps healthcare providers assess the overall health status of a patient. Clinical characteristics encompassed activities of daily living (ADL) and number of admissions to medical wards within the year before death. ADL refers to the basic tasks of daily self-care performed by patients on an independent basis. These tasks are essential for personal care and include eating, bathing, dressing, toileting, transferring, continence and mobility. The total score ranges from 0 to 100, with higher scores indicating greater independence. Laboratory data, medication use and patient mobility information were collected for the 6 months before death. Drug consumption was calculated using the defined daily dose (DDD). Patients who had used first-generation antipsychotics (FGAs) were grouped as FGA users. The daily consumption of FGAs was computed based on the DDD. The same rules apply to second-generation antipsychotics (SGAs), clozapine, benzodiazepines (BZDs), anticholinergics and mood stabilisers. Causes of death were classified into six groups: cardiovascular disease, cancer, pneumonia, choking, other infections and the remainder.

### Statistical analysis

Continuous data are expressed as means with standard deviation, and categorical variables are presented as frequencies (percentages). Independent *t*-tests were used for comparing continuous variables, whereas Pearson's *χ*^2^ tests or Fisher's exact tests were used to compare categorical variables between longevity and control groups. Effect sizes with 95% confidence intervals were expressed with Cohen's *d* and odds ratios for continuous and categorical variables, respectively. If the distribution of continuous variables was skewed, the Mann–Whitney *U*-test was conducted. In this situation, the median with the interquartile range (IQR) was provided. To investigate the predictors of the longevity of patients with schizophrenia receiving long-term care, a multivariate logistic regression analysis using the forward selection method was performed, with longevity serving as the dependent variable. Logistic regression is suited for this study because of its effectiveness in handling binary outcomes, robustness with small sample sizes, flexibility with different types of predictor variables and interpretability of results. To inspect the shared traits among individuals with longevity, cluster analysis was employed to identify naturally occurring groups within the study participants, utilising the significant predictors recognised in the logistic regression analysis. The comparison of age at death and the proportion of longevity between clusters were conducted using independent *t*-tests and *χ*^2^ tests, respectively. Using cluster analysis on the significant predictors identified by logistic regression can reveal natural groupings, enhance data interpretation, validate findings, reveal outliers and provide clear visual representations. The two-step method was used, as it combines the strengths of both *k*-means and hierarchical clustering. This method was suitable for this study because of its ability to handle mixed data types, automatically determine the number of clusters and offer scalability, robustness and interpretability. These benefits make cluster analysis a valuable complementary technique in the research process. In an effort to address confounders, relevant variables as covariates were included in the logistic regression model to evaluate the effect of the identified predictors, accounting for the impact of other variables. All statistical tests were two-sided, with a *P*-value < 0.05 considered statistically significant. For the comparisons of age at death among different causes of mortality, the Mann–Whitney *U*-test with the Bonferroni multiple-adjustment method was applied to account for multiple comparisons. A *P*-value < 0.0125 was defined as statistically significant. Statistical analysis was executed using SPSS for Windows version 21.0 (IBM, Armonk, NY, USA).

### Ethics and consent statement

The authors assert that all procedures contributing to this work comply with the ethical standards of the relevant national and institutional committees on human experimentation and with the Helsinki Declaration of 1975, as revised in 2008. All procedures involving human participants were approved by the institutional review board (IRB) of Yuli Hospital (protocol code YLH-IRB-10614 and date of approval: 24 November 2017). The participants of this study are deceased; exemption from obtaining informed consent was granted by the IRB. To ensure research ethics, the anonymity of patients remains preserved.

## Results

### Distribution of variables

The average age at death for all 138 participants was 68.00 years (s.d. = 11.63), with the 45 participants in the longevity group averaging 80.53 years (s.d. = 4.55). The distribution of variables by longevity and control groups is summarised in Table 2. The longevity group had significantly older age at onset (Cohen's *d* = 0.55, 95% CI 0.18–0.91, *P* = 0.010), longer length of stay (Cohen's *d* = 0.63, 95% CI 0.27–1.00, *P* < 0.001), more admissions to general medical wards (Cohen's *d* = 0.42, 95% CI 0.06–0.78, *P* = 0.024) and lower daily dosages of antipsychotic medications (Cohen's *d* = −0.70, 95% CI −1.07 to −0.33, *P* < 0.001), clozapine (Cohen's *d* = −0.61, 95% CI −0.97 to −0.25, *P* < 0.001) and BZDs (Cohen's *d* = −0.35, 95% CI −0.70–0.01, *P* = 0.029). There were no significant differences between the two groups for either generation of antipsychotics, the CCI, the prevalence of diabetes mellitus or hyperlipidaemia. The longevity group exhibited a higher proportion of males compared to the control group (odds ratio = 2.63, 95% CI 0.93–7.45, *P* = 0.047). Notably, this group demonstrated lower scores for ADL (Cohen's *d* = −0.97, 95% CI −1.32 to −0.58, *P* < 0.001), increased occurrences of poor mobility (odds ratio = 5.15, 95% CI 2.17–12.24, *P* < 0.001) and a higher prevalence of hypertension diagnoses (odds ratio = 3.11, 95% CI 1.48–6.54, *P* = 0.002). Regarding causes of death, pneumonia was the leading natural cause of death (*N* = 56, 40.6%), followed by cardiovascular disease (*N* = 29, 21.0%) and cancer (*N* = 21, 15.2%). Choking was the primary cause of unnatural deaths (*N* = 12, 8.7%). The longevity group featured a higher mortality rate attributed to pneumonia (odds ratio = 3.31, 95% CI 1.58–6.94, *P* = 0.001), along with a decreased mortality rate from cancer (odds ratio = 0.18, 95% CI 0.04–0.82, *P* = 0.010) and incidents of choking.

**Table 2:** Distribution of continuous and categorical variables for longevity and control groups

Variables	All (*N* = 138)	Longevity group (*N* = 45)	Control group (*N* = 93)	
	Mean (s.d.)	Mean (s.d.)	Mean (s.d.)	*P*^a^	Cohen's *d* (95% CI)
Age at death	68.00 (11.63)	80.53 (4.55)	61.94 (8.80)	<0.001	2.42 (1.96−2.88)
Age at onset	24.36 (8.42)	27.36 (10.22)	22.90 (7.01)	0.010	0.55 (0.18−0.91)
Length of stay	25.54 (13.74)	31.18 (13.25)	22.82 (13.20)	<0.001	0.63 (0.27−1.00)
ADL	61.28 (39.10)	38.36 (37.13)	72.37 (35.15)	<0.001	−0.97 (−1.32 to −0.58)
Number of admissions to general medical wards	2.88 (2.95)	3.69 (3.05)	2.48 (2.83)	0.024	0.42 (0.06–0.78)
Number of admissions to acute psychiatric wards	0.11 (0.38)	0.07 (0.25)	0.13 (0.42)	0.363	−0.16 (−0.52 to 0.20)
Albumin	4.43 (13.85)	2.93 (0.62)	5.21 (17.07)	0.390	−0.16 (−0.52 to 0.19)
Antipsychotic DDD	0.56 (0.62)	0.27 (0.35)	0.68 (0.67)	<0.001	−0.70 (−1.07 to −0.33)
Mood stabiliser DDD	0.06 (0.34)	0.02 (0.09)	0.08 (0.40)	0.358	−0.18 (−0.54 to 0.18)
FGA DDD	0.12 (0.33)	0.05 (0.18)	0.15 (0.38)	0.060	−0.30 (−0.66 to 0.05)
SGA DDD	1.71 (14.22)	0.22 (0.34)	2.32 (16.89)	0.457	−0.15 (−0.51 to 0.21)
Clozapine DDD	0.10 (0.25)	0.00 (0.01)	0.14 (0.28)	<0.001	−0.61 (−0.97 to −0.25)
BZD DDD	0.29 (0.50)	0.17 (0.31)	0.34 (0.56)	0.029	−0.35 (−0.70 to 0.01)
Anticholinergic DDD	0.12 (0.18)	0.08 (0.16)	0.14 (0.18)	0.106	−0.35 (−0.70 to 0.01)
CCI	1.38 (1.40)	1.38 (1.48)	1.39 (1.36)	0.971	−0.01 (−0.36 to 0.35)
	*N* (%)	*N* (%)	*N* (%)	*P*^b^	Odds ratio (95% CI)
Male	110 (79.9)	40 (88.9)	70 (75.3)	0.047	2.63 (0.93–7.45)
Causes of death					
Cardiovascular disease	29 (21.0)	12 (26.7)	17 (18.3)	0.181	1.63 (0.70–3.78)
Cancer	21 (15.2)	2 (4.4)	19 (20.4)	0.010	0.18 (0.04–0.82)
Pneumonia	56 (40.6)	27 (60.0)	29 (31.2)	0.001	3.31 (1.58–6.94)
Choking	12 (8.7)	0 (0.0)	12 (12.9)	0.007	–
Poor mobility	81 (58.7)	37 (82.2)	44 (47.3)	<0.001	5.15 (2.17–12.24)
FGA user	22 (17.9)	5 (13.9)	17 (19.5)	0.321	0.66 (0.23–1.96)
SGA user	81 (65.9)	20 (55.6)	61 (70.1)	0.091	0.53 (0.24–1.19)
Clozapine user	24 (19.5)	1 (2.8)	23 (26.4)	0.001	0.08 (0.01–0.61)
Diabetes mellitus	31 (22.5)	9 (20.0)	22 (23.7)	0.401	0.81 (0.34–1.93)
Hypertension	49 (35.5)	24 (53.3)	25 (26.9)	0.002	3.11 (1.48–6.54)
Hyperlipidaemia	16 (11.6)	4 (8.9)	12 (12.9)	0.351	0.66 (0.20–2.17)

ADL, activities of daily living; DDD, defined daily dose; FGA, first-generation antipsychotic; SGA, second-generation antipsychotic; BZD, benzodiazepine; CCI, Charlson comorbidity index.

a. Independent *t*-test.

b. *χ*^2^ test.

### Predictors of longevity

Table 3 summarises the logistic regression analysis, revealing the predictors significantly associated with longevity. Older age at onset was associated with greater longevity, with an adjusted odds ratio (aOR) of 1.09 (95% CI 1.02–1.17, *P* = 0.009). Similarly, longer length of stay was positively associated with longevity, with aOR = 1.10 (95% CI 1.04–1.15, *P* < 0.001). In contrast, the ADL score within 1 year of death had an aOR of 0.98 (95% CI 0.96–0.99, *P* = 0.001), indicating that a lower ADL score was associated with a higher likelihood of living longer. Moreover, the findings displayed a significant association between longevity and hypertension (aOR = 3.35, 95% CI 1.11–10.13, *P* = 0.032).

**Table 3:** Multivariate logistic regression analyses for association of predictors and longevity

Predictors	Adjusted odds ratio	95% CI	*P*
Age at onset	1.09	1.02–1.17	0.009
Length of stay	1.10	1.04–1.15	<0.001
ADL	0.98	0.96–0.99	0.001
Hypertension	3.35	1.11–10.13	0.032

ADL, activities of daily living.

### Naturally occurring groups within the study participants

There were two groups identified by cluster analysis based on the predictors of longevity. They exhibited distinct demographic and health-related patterns. Group 1 (*n* = 49) had an average age at onset of 26.92 years (s.d. = 9.62) and a mean residence duration of 25.00 years (s.d. = 14.15). The ADL for Group 1 had a mean score of 54.51 (s.d. = 39.97), with hypertension prevalent in all cases (100.0%). The average age at death in this group was 72.63 years (s.d. = 9.53), and the longevity rate was 49.0% (*n* = 24). In contrast, Group 2 (*n* = 89) had an average age at onset of 22.94 years (s.d. = 7.36) and a mean residence duration of 25.84 years (s.d. = 13.59). The ADL mean score for Group 2 was 65.00 (s.d. = 38.32), and no cases of hypertension were observed (0.0%). The average age at death in Group 2 was 65.45 years (s.d. = 11.93), with a longevity rate of 23.6% (*n* = 21). Group 1 featured a significantly higher average age at death (*P* < 0.001) and a significantly higher longevity rate (*P* = 0.002). To determine if there is a difference in the number of samples between the two classifications (longevity or not and clustering groups by cluster analysis), McNemar's test for paired samples was employed to evaluate their marginal homogeneity. The result of McNemar's test showed *P* = 0.659 > 0.05, indicating that the difference in the number of samples between the two classifications is not significant (Table 4).

**Table 4:** Naturally occurring groups identified by cluster analysis within the study participants

Group	Variables for clustering				Age at death mean (s.d.)	Longevity *N* (%)	McNemar's test
	Age at onset mean (s.d.)	Length of stay mean (s.d.)	ADL mean (s.d.)	Hypertension *N* (%)			
Group 1 (*n* = 49)	26.92 (9.62)	25.00 (14.15)	54.51 (39.97)	49 (100.0)	72.63 (9.53)	24 (49.0)	
Group 2 (*n* = 89)	22.94 (7.36)	25.84 (13.59)	65.00 (38.32)	0 (0.0)	65.45 (11.93)	21 (23.6)	
*P*^a^					<0.001	0.002	0.659

ADL, activities of daily living.

a. *P*-value for comparison among groups by independent *t*-test for age at death, *χ*^2^ test for longevity and McNemar's test for paired samples to evaluate their marginal homogeneity.

### Comparing causes of death by age

**Figure 1:**
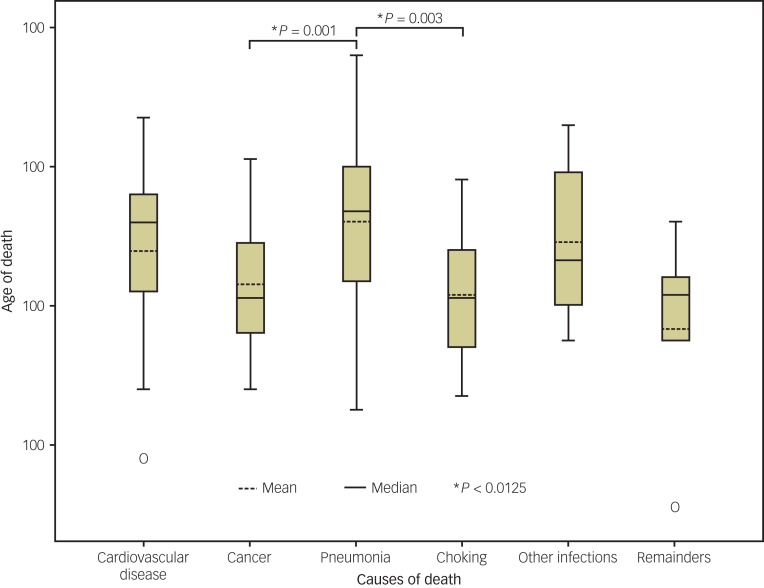
Interquartile box plots for age at death among various causes of death. Compared to pneumonia fatalities, the ages at death were notably lower for cancer and choking fatalities. However, there was no significant difference in the ages at death between pneumonia fatalities and those from cardiovascular disease or other infections.

There were significant variations in the age at death among different causes of death, as shown in Table 5 and Fig. 1. When compared to pneumonia fatalities (mean = 68.0, s.d. = 12.13, median = 72), the age at death was significantly lower for cancer fatalities (mean = 63.2, s.d. = 8.04, median = 61, *P* = 0.001) and choking fatalities (mean = 61.4, s.d. = 9.56, median = 61, *P* = 0.003). However, no significant difference in the age at death was observed between pneumonia fatalities and those from cardiovascular disease or other infections.

**Table 5:** Comparing age at death among various causes, using pneumonia fatalities as the reference

Causes of death	Age at death				*P*
	Mean	s.d.	Median	IQR	
Pneumonia (*N* = 56)	72.0	11.35	73.5	63.3–80	–
Cancer (*N* = 21)	63.2	8.04	61.0	56–69	0.001
Choking (*N* = 12)	61.4	9.56	61.0	54–69.5	0.003
Cardiovascular disease (*N* = 29)	68.0	12.13	72.0	59–76	0.122
Other infections (*N* = 14)	69.2	10.55	66.5	59.8–80	0.370
Remainders^a^ (*N* = 6)	57.5	14.10	61.5	49–66	N/A

IQR, interquartile range.

a. Remainders included five organ failures, three cerebrovascular events and one suicide. A statistical comparison was not applicable because of the heterogeneity of the remaining causes of death.

## Discussion

### Main findings

This is the first study aimed at identifying predictors of longevity in long-term care facility residents with schizophrenia. Individuals in the longevity group had a higher male predominance, poorer mobility, older age at onset, longer length of stay, more admissions to general medical wards, lower ADL scores and a higher prevalence of hypertension. They received lower dosages of psychotropics and had higher mortality rates attributed to pneumonia. Older age at onset, longer length of stay, lower ADL scores and having a diagnosis of hypertension were significant predictors of longevity. Cluster analysis identified two patient groups, suggesting that having poorer health indicators did not necessarily result in shorter lives. With regards to causes of death, it was found that pneumonia fatalities were linked to a higher age at death, compared to those from cancer or choking.

### Variables associated with longevity

Male predominance in the longevity group is inconsistent with previous findings showing higher excess mortality rates among men with schizophrenic spectrum disorders.^21^ The discrepancy may be attributed to the protective environment provided by long-term care. Risk factors such as tobacco dependence, substance use disorders,^22^ lower levels of physical activity and non-adherence to physical activity guidelines^4^ are more prevalent among men but can be more effectively controlled and managed within the therapeutic milieu, potentially reducing the risk of premature death. This could also support the observed gender difference that men experience faster improvements in life expectancy than women if appropriate preventive health measures are implemented.^9^ Mobility impairments in patients with schizophrenia may result from Parkinsonism induced by antipsychotic medications and a reduced physiological reserve because of physical illnesses. These impairments increase the risk of falls, the second leading cause of unintentional injury deaths globally.^23^ However, research on Parkinson's disease has shown that addressing movement dysfunction can help patients regain mobility, significantly improving life expectancy.^24^ Implementing appropriate interventions to address poor mobility and resulting falls^25^ may reduce excess mortality in this population.

Antipsychotics remain a cornerstone in the treatment of schizophrenia. Patients not taking antipsychotics had the highest risk of death.^7^ However, gradual dose reduction may be a viable option for elderly patients. An international expert consensus recommended a 50% lower median daily oral antipsychotic dose for elderly patients.^26^ Age-related decline in hepatic drug metabolism may increase clozapine concentration and related side-effects, potentially limiting its protective effect in reducing long-term mortality.^27^ In addition, BZD use has been associated with increased mortality risk in patients with schizophrenia.^8^ The present results are consistent with previous studies suggesting that tailoring the medication type and dose based on age may increase lifespan.

### Predictors of longevity

Age at onset is crucial regarding the prognosis of schizophrenia, but there is conflicting evidence on the relationship between age at onset and lifespan. Patients with very-late-onset schizophrenia-like psychosis were at a higher risk of death than those with earlier onset, mainly because of physical comorbidities and accidents.^28^ However, this study found that older age at onset predicts longevity in individuals with schizophrenia, possibly because of better illness insight^29^ leading to more cooperation with medical intervention^30^ under long-term care. The present study observed a potential link between decreased daily living activity and a hypertension diagnosis, with increased longevity among long-term care residents with schizophrenia. The finding contradicts the prevailing understanding; nonetheless, it can be reasonably explained in the context of medical team-based psychiatric long-term care. When patients exhibit poor participation in daily living activities, staff will take the initiative to assist and provide comprehensive health promotion activities. Psychiatrists and other physicians are responsible for diagnosing and treating systemic diseases to ensure prompt medical attention. Conversely, community-dwelling patients with mental illness who have poor ADL scores or are diagnosed with hypertension are more susceptible to complications and death if they cannot access medical care. As such, a decline in ADL is a poor prognostic indicator for patients living in the community, but serves as a critical warning sign for those receiving long-term care. A recent comprehensive nationwide study^6^ examined the impact of schizophrenia on mortality and its relationship with variables influencing life expectancy. They found that individuals with schizophrenia but no Charlson comorbidities had the highest increase in mortality risk for natural causes and suicide. This is because physical illnesses in patients with schizophrenia are common but often underdetected and undertreated, which may explain the significant mortality difference compared to healthier individuals without schizophrenia. Early detection of poor physical health in these patients allows for timely intervention and potentially extends their lifespan.

Patients with schizophrenia have higher mortality risks because of reduced medical utilisation. Long-term care offers not only supportive housing but also individualised medical care. Therefore, the length of stay represents not just the duration of the stay but can also be employed as a proxy for the amount of medical care. A retrospective study demonstrated that longer intensive care unit (ICU) stays for major psychiatric disorder patients correlated with lower mortality rates,^31^ highlighting the potential role of appropriate medical treatment in extending lifespan by reducing short-term mortality. Present findings support previous suggestions that interventions targeting proper healthcare usage and treatment of medical comorbidities among individuals with severe mental illness could help reduce the mortality gap caused by physical illness.^32^ Long-term stays in hospital for schizophrenia have declined because of the availability of alternative treatments and a shift towards community-based care. Nevertheless, some individuals with severe symptoms or a lack of community support may still require institutionalisation.^33^ This study found that receiving longer medical team-based long-term residential care can lead to more individualised medical treatments and increased longevity. However, it is important to consider the similar potential risks associated with prolonged stays in hospital, such as social isolation and a decrease in personal autonomy.^34^ Treatment decisions should consider the individual's needs and preferences and the availability of community-based resources.

### Shared traits among individuals with longevity

Research on human ageing provides several empirical strategies that favour an increased healthy lifespan, including eating in moderation, regular exercise, purposeful living and strong social support systems. While some studies propose that certain biomarkers may forecast longevity, many are speculative and have limitations. There is unlikely to be a single biomarker predictive of long life.^35^ This study used cluster analysis to identify two distinct groups among patients with schizophrenia, challenging conventional assumptions about longevity. Despite poorer health indicators in Group 1, they lived longer and had higher longevity rates compared to Group 2, which had better functional abilities but lived shorter lives. Other than the benefit of early management of health problems while receiving regular hypertension treatment,^36^ the age at onset may be important in mediating the lifespan of patients with schizophrenia. This supposition is based on the evidence that the same apolipoprotein E genetic polymorphism may be linked to the age at onset in schizophrenia and the likelihood of surviving to advanced age in the general population.^19, 37^

### Causes of death

Pneumonia was the major cause of mortality in the longevity group. Cancer and choking had lower median ages of death relative to pneumonia. By promoting greater adherence to antipsychotic medication in long-term care settings, the risk of discontinuation of cardiometabolic medications was lowered.^38^ Furthermore, modifiable cardiovascular risk factors, such as tobacco use, substance misuse, an unhealthy diet, a lack of exercise and weight gain, could also be effectively addressed. All of these explained the decreased cardiovascular mortality in the longevity group. Cancer patients in this population had a median death age of 61, but the median age of cancer deaths in Taiwan during these 3 years was 69 years old.^39^ Given the potential healthcare disparities indicated by the elevated cancer mortality-to-incidence ratio (MIR) in patients with schizophrenia,^40^ individuals already integrated into the healthcare system may experience an increased risk of early cancer mortality because of mistrust of the medical system and inadequate familial support.^41^ Dysphagia is a common disorder in patients with schizophrenia and may contribute to choking deaths.^42^ It is postulated that the increased susceptibility to choking in younger patients may be attributed to behavioural changes related to schizophrenia, including eating too quickly or taking inappropriately large boluses of food.^43^ Conversely, older patients commonly need feeding assistance, allowing caregivers to optimise the rate and quantity of food intake. Moreover, the alimentary composition can also be appropriately modified based on the severity of extrapyramidal symptoms. There was only one death caused by suicide in this sample. Aside from actively and regularly monitoring suicide risk within the facility, suicide is more common among young and recently diagnosed patients.^6,44^ Our research primarily involved older individuals with chronic schizophrenia, which explains the low suicide rate in this study.

### Strengths and limitations

The main strength of this study is that it was conducted at the hospital level, allowing for the collection of individualised clinical information not available in population-based registry databases. Another strength is that the NHIRD was used to estimate the age of the 90th survival percentile, which enabled defining longevity based on national data. Combining the advantages of these two data-sets improves the validity and generalisability of our findings. In addition, this study population is highly representative of patients with schizophrenia in long-term care. Previous studies on long-term care for schizophrenia have been limited by sample size.^45^ The psychiatric facility is one of the two largest long-term care facilities in Taiwan, providing care for the majority of the country's patients with schizophrenia who require long-term care. During the study period, there was an average of up to 2368 patients from various parts of Taiwan who had been long-term residents of this facility, providing a substantial sample source for this research.

There are some limitations to our study. First, our investigation was conducted at a single site, which may compromise the representativeness of the results. Although the facility provides long-term care services for patients with schizophrenia from across Taiwan, the generalisability of our findings to other settings or populations remains uncertain. To mitigate this limitation, future studies should aim to include multiple sites with diverse demographic and clinical characteristics to enhance the representativeness of the results. Second, the study spanned only 3 years and included 138 mortality cases. The relatively short duration and modest sample size may affect the robustness of the findings. A larger sample size and an extended follow-up period are necessary to increase the statistical power and reliability of the results. Future research should consider longer-term studies with more extensive data-sets to verify the findings and provide more definitive conclusions. Third, the cross-sectional design of the study restricts the ability to establish causality. While we identified associations between certain variables and outcomes, a cause-and-effect relationship cannot be inferred. Longitudinal cohort studies are required to track changes over time and determine causal links. Such studies will provide a more comprehensive understanding of the factors influencing longevity in patients with schizophrenia receiving long-term care. In summary, while our study provides valuable insights, addressing these limitations through future research is essential for validating and expanding upon our findings. By incorporating multi-site data, larger sample sizes, extended follow-up periods and longitudinal designs, future studies can overcome these limitations and contribute more robust and generalisable knowledge to the field.

The study results suggest that an older age at onset, longer length of stay, lower ADL scores and having a diagnosis of hypertension may serve as potential prognostic markers of longevity for schizophrenia in medical team-based psychiatric long-term care settings. Management addressing modifiable risk factors is effective in enhancing life expectancy in schizophrenia, especially for males. Considering the common characteristics among individuals with longevity, the age at onset may play an important role in reflecting the lifespan of schizophrenia. In addition, pneumonia remains a leading cause of mortality in this population. While these results seem contrary to conventional understanding, they can be clarified through the implementation of comprehensive long-term care involving coordination between mental health and general healthcare systems. Further research is needed to validate and extend these results and to determine the optimal balance between long-term residential care and community-based care for individuals with schizophrenia in later life.

## Data Availability

The data that support the findings of this study are available from the corresponding author, T.H., upon reasonable request.

## References

[1] Marder SR, Cannon TD. Schizophrenia. N Engl J Med 2019; 381(18): 1753–61. 31665579 10.1056/NEJMra1808803

[2] Holt RI. The prevention of diabetes and cardiovascular disease in people with schizophrenia. Acta Psychiatr Scand 2015; 132(2): 86–96. 25976975 10.1111/acps.12443

[3] Kuo CJ, Yang SY, Liao YT, Chen WJ, Lee WC, Shau WY, et al. Second-generation antipsychotic medications and risk of pneumonia in schizophrenia. Schizophr Bull 2013; 39(3): 648–57. 22282455 10.1093/schbul/sbr202 PMC3627761

[4] Vancampfort D, Firth J, Schuch FB, Rosenbaum S, Mugisha J, Hallgren M, et al. Sedentary behavior and physical activity levels in people with schizophrenia, bipolar disorder and major depressive disorder: a global systematic review and meta-analysis. World Psychiatry 2017; 16(3): 308–15. 28941119 10.1002/wps.20458 PMC5608847

[5] Olfson M, Gerhard T, Huang C, Crystal S, Stroup TS. Premature mortality among adults with schizophrenia in the United States. JAMA Psychiatry 2015; 72(12): 1172–81. 26509694 10.1001/jamapsychiatry.2015.1737

[6] Cheng CM, Chang WH, Tsai SJ, Li CT, Tsai CF, Bai YM, et al. Risk of all-cause and suicide death in patients with schizophrenia: an entire-population longitudinal study in Taiwan. J Clin Psychiatry 2023; 84(6): 22m14747. 10.4088/JCP.22m14747 37707313

[7] Torniainen M, Mittendorfer-Rutz E, Tanskanen A, Björkenstam C, Suvisaari J, Alexanderson K, et al. Antipsychotic treatment and mortality in schizophrenia. Schizophr Bull 2015; 41(3): 656–63. 25422511 10.1093/schbul/sbu164 PMC4393693

[8] Tiihonen J, Suokas JT, Suvisaari JM, Haukka J, Korhonen P. Polypharmacy with antipsychotics, antidepressants, or benzodiazepines and mortality in schizophrenia. Arch Gen Psychiatry 2012; 69(5): 476–83. 22566579 10.1001/archgenpsychiatry.2011.1532

[9] Meyer AC, Drefahl S, Ahlbom A, Lambe M, Modig K. Trends in life expectancy: did the gap between the healthy and the ill widen or close? BMC Med 2020; 18(1): 41. 32192480 10.1186/s12916-020-01514-z PMC7082956

[10] Lee EE, Liu J, Tu X, Palmer BW, Eyler LT, Jeste DV. A widening longevity gap between people with schizophrenia and general population: a literature review and call for action. Schizophr Res 2018; 196: 9–13. 28964652 10.1016/j.schres.2017.09.005 PMC5955767

[11] Laursen TM, Nordentoft M, Mortensen PB. Excess early mortality in schizophrenia. Ann Rev Clinical Psychol 2014; 10: 425–48. 24313570 10.1146/annurev-clinpsy-032813-153657

[12] Lêng CH, Chou MH, Lin SH, Yang YK, Wang JD. Estimation of life expectancy, loss-of-life expectancy, and lifetime healthcare expenditures for schizophrenia in Taiwan. Schizophr Res 2016; 171(1–3): 97–102. 26811230 10.1016/j.schres.2016.01.033

[13] Correll CU, Solmi M, Croatto G, Schneider LK, Rohani-Montez SC, Fairley L, et al. Mortality in people with schizophrenia: a systematic review and meta-analysis of relative risk and aggravating or attenuating factors. World Psychiatry 2022; 21(2): 248–71. 35524619 10.1002/wps.20994 PMC9077617

[14] Meesters PD. New horizons in schizophrenia in older people. Age Ageing 2023; 52(9): afad161. 37725971 10.1093/ageing/afad161

[15] Kahn RS, Sommer IE, Murray RM, Meyer-Lindenberg A, Weinberger DR, Cannon TD, et al. Schizophrenia. Nat Rev Dis Primers 2015; 1: 15067. 27189524 10.1038/nrdp.2015.67

[16] Chien IC, Chou YJ, Lin CH, Bih SH, Chou P, Chang HJ. Prevalence and incidence of schizophrenia among national health insurance enrollees in Taiwan, 1996–2001. Psychiatry Clin Neurosci 2004; 58(6): 611–8. 15601385 10.1111/j.1440-1819.2004.01311.x

[17] Cohen CI, Meesters PD, Zhao J. New perspectives on schizophrenia in later life: implications for treatment, policy, and research. Lancet Psychiatry 2015; 2(4): 340–50. 26360087 10.1016/S2215-0366(15)00003-6

[18] Lee P-C, Wang JT-H, Chen T-Y, Peng C-H. Digital Health Care in Taiwan: Innovations of National Health Insurance. Springer Nature, 2022.

[19] Deelen J, Evans DS, Arking DE, Tesi N, Nygaard M, Liu X, et al. A meta-analysis of genome-wide association studies identifies multiple longevity genes. Nat Commun 2019; 10(1): 3669. 31413261 10.1038/s41467-019-11558-2 PMC6694136

[20] Charlson ME, Pompei P, Ales KL, MacKenzie CR. A new method of classifying prognostic comorbidity in longitudinal studies: development and validation. J Chronic Dis 1987; 40(5): 373–83. 3558716 10.1016/0021-9681(87)90171-8

[21] Meesters PD, Comijs HC, Smit JH, Eikelenboom P, de Haan L, Beekman AT, et al. Mortality and its determinants in late-life schizophrenia: a 5-year prospective study in a Dutch catchment area. Am J Geriatr Psychiatry 2016; 24(4): 272–7. 26796925 10.1016/j.jagp.2015.09.003

[22] Hunt GE, Large MM, Cleary M, Lai HMX, Saunders JB. Prevalence of comorbid substance use in schizophrenia spectrum disorders in community and clinical settings, 1990–2017: systematic review and meta-analysis. Drug Alcohol Depend 2018; 191: 234–58. 30153606 10.1016/j.drugalcdep.2018.07.011

[23] World Health Organization (WHO). *Falls 2021*. WHO, 2021 (https://www.who.int/news-room/fact-sheets/detail/falls).

[24] Rodriguez-Oroz MC, Moro E, Krack P. Long-term outcomes of surgical therapies for Parkinson's disease. Mov Disord 2012; 27(14): 1718–28. 23208668 10.1002/mds.25214

[25] Tsai MT, Lee SM, Chen HK, Wu BJ. Association between frailty and its individual components with the risk of falls in patients with schizophrenia spectrum disorders. Schizophr Res 2018; 197: 138–43. 29395605 10.1016/j.schres.2018.01.023

[26] Gardner DM, Murphy AL, O'Donnell H, Centorrino F, Baldessarini RJ. International consensus study of antipsychotic dosing. Am Journal Psychiatry 2010; 167(6): 686–93. 20360319 10.1176/appi.ajp.2009.09060802

[27] Vermeulen JM, van Rooijen G, van de Kerkhof MPJ, Sutterland AL, Correll CU, de Haan L. Clozapine and long-term mortality risk in patients with schizophrenia: a systematic review and meta-analysis of studies lasting 1.1–12.5 years. Schizophr Bull 2019; 45(2): 315–29. 29697804 10.1093/schbul/sby052 PMC6403051

[28] Talaslahti T, Alanen HM, Hakko H, Isohanni M, Häkkinen U, Leinonen E. Patients with very-late-onset schizoprhenia-like psychosis have higher mortality rates than elderly patients with earlier onset schizophrenia. Int J Geriatr Psychiatry 2015; 30(5): 453–9. 24990229 10.1002/gps.4159

[29] Tariku M, Demilew D, Fanta T, Mekonnen M, Abebaw Angaw D. Insight and associated factors among patients with schizophrenia in mental specialized hospital, Ethiopia, 2018. Psychiatry J 2019; 2019: 2453862. 31915674 10.1155/2019/2453862 PMC6930763

[30] Droulout T, Liraud F, Verdoux H. Relationships between insight and medication adherence in subjects with psychosis. L'Encephale 2003; 29(5): 430–7. 14615692

[31] King DA, Hussein E, Shochet GE, Bar-Lavie YP. Admission rate of patients with major psychiatric disorders to the intensive care unit. Am J Crit Care 2020; 29(6): 480–3. 33130867 10.4037/ajcc2020934

[32] Launders N, Hayes JF, Price G, Marston L, Osborn DPJ. The incidence rate of planned and emergency physical health hospital admissions in people diagnosed with severe mental illness: a cohort study. Psychol Med 2023; 53(12): 5603–14. 36069188 10.1017/S0033291722002811 PMC10482715

[33] Uggerby P, Nielsen RE, Correll CU, Nielsen J. Characteristics and predictors of long-term institutionalization in patients with schizophrenia. Schizophr Res 2011; 131(1–3): 120–6. 21458239 10.1016/j.schres.2011.03.001

[34] Smith P, Nicaise P, Giacco D, Bird VJ, Bauer M, Ruggeri M, et al. Use of psychiatric hospitals and social integration of patients with psychiatric disorders: a prospective cohort study in five European countries. Soc Psychiatry psychiatr Epidemiol 2020; 55(11): 1425–38. 32409885 10.1007/s00127-020-01881-1 PMC7578147

[35] Pignolo RJ. Exceptional human longevity. Mayo Clin Proc 2019; 94(1): 110–24. 30545477 10.1016/j.mayocp.2018.10.005

[36] Starfield B, Shi L, Macinko J. Contribution of primary care to health systems and health. Milbank Q 2005; 83(3): 457–502. 16202000 10.1111/j.1468-0009.2005.00409.x PMC2690145

[37] Akanji AO, Ohaeri JU, Al-Shammri SN, Fatania HR. Apolipoprotein E polymorphism and clinical disease phenotypes in arab patients with schizophrenia. Neuropsychobiology 2009; 60(2): 67–72. 19752580 10.1159/000236446

[38] Solmi M, Tiihonen J, Lähteenvuo M, Tanskanen A, Correll CU, Taipale H. Antipsychotics use is associated with greater adherence to cardiometabolic medications in patients with schizophrenia: results from a nationwide, within-subject design study. Schizophr Bull 2022; 48(1): 166–75. 34286338 10.1093/schbul/sbab087 PMC8781351

[39] Taiwan Ministry of Health and Welfare (MOHW). *Cause of Death Statistics*. MOHW (https://www.mohw.gov.tw/np-128-2.html).

[40] Cheng CS, Chen WY, Chang HM, Pan CH, Su SS, Tsai SY, et al. Unfavorable cancer mortality-to-incidence ratios in patients with schizophrenia: a nationwide cohort study in Taiwan, 2000–2019. Acta Psychiatr Scand 2023; 148(4): 347–58. 37607118 10.1111/acps.13604

[41] Gonzalez-Rodriguez A, Labad J, Seeman MV. Schizophrenia and cancer. Curr Opin Support Palliat Care 2020; 14(3): 232–8. 32701859 10.1097/SPC.0000000000000512

[42] Regan J, Sowman R, Walsh I. Prevalence of Dysphagia in acute and community mental health settings. Dysphagia 2006; 21(2): 95–101. 16763936 10.1007/s00455-006-9016-9

[43] Fioritti A, Giaccotto L, Melega V. Choking incidents among psychiatric patients: retrospective analysis of thirty-one cases from the west Bologna psychiatric wards. Can J Psychiatry 1997; 42(5): 515–20. 9220116 10.1177/070674379704200509

[44] Lin JJ, Liang FW, Li CY, Lu TH. Leading causes of death among decedents with mention of schizophrenia on the death certificates in the United States. Schizophr Res 2018; 197: 116–23. 29395608 10.1016/j.schres.2018.01.011

[45] Lavaud P, McMahon K, Sanchez Rico M, Hanon C, Alvarado JM, de Raykeer RP, et al. Long-term care utilization within older adults with schizophrenia: associated factors in a multicenter study. Psychiatry Res 2022; 308: 114339. 34963089 10.1016/j.psychres.2021.114339

